# Comparative study of new imaging technologies for the diagnosis of glaucoma: design and conduct of a multi-centre diagnostic accuracy study

**DOI:** 10.1186/1745-6215-14-S1-O45

**Published:** 2013-11-29

**Authors:** Katie Banister, Jonathan Cook, Craig Ramsay, Jennifer Burr, Rodolfo Hernández, Kirsty McCormack, Rupert Bourne, Mark Batterbury, David Garway-Heath, Augusto Azuara-Blanco

**Affiliations:** 1 University of Aberdeen, Aberdeen, UK; 2 University of St Andrews, St Andrews, UK; 3 Hinchingbrooke Hospital NHS Trust, Huntingdon, UK; 4 Royal Liverpool and Broadgreen University Hospitals NHS Trust, Liverpool, UK; 5 Moorfields Eye Hospital NHS Foundation Trust, London, UK; 6 Queen's University Belfast, Belfast, UK

## Purpose

The detection and diagnosis of glaucoma is challenging for health professionals. In the UK, approximately 45% of patients are discharged from secondary care after one visit.

Automated imaging technologies are easy to perform and could potentially be used by trained technicians as triage tests for glaucoma diagnosis.

The GATE study aims to compare the diagnostic performance of three technologies, Heidelberg Retina Tomograph (HRT-III), Scanning laser polarimetry (GDx-ECC) and Optical Coherence Tomography (Spectralis), as triage tests in secondary care for glaucoma diagnosis.

## Method

### Design

Diagnostic accuracy study, comparing 3 imaging techniques for glaucoma diagnosis.

### Population

Adult patients, newly referred from community to hospital eye services for suspected glaucoma.

### Reference standard

Comprehensive clinical examination by experienced consultant ophthalmologist, including fundus examination and visual field tests.

### Sample size

954, each imaged using all three technologies.

### Setting

NHS secondary care, UK.

### Outcomes

Diagnostic performance measures, economic outcomes.

### Data collection

Data uploaded at site via secure web-based data-collection system.

## Results

Recruitment commenced April 2011. To date, 874 participants have been enrolled from five UK hospitals. GATE is an on-going research study and will be completed in November 2013.

## Discussion

Conducting a multicentre diagnostic accuracy study in ophthalmology is challenging. Problems which were overcome are grouped into: difficulties in site set-up, consensus in agreeing a reference standard and agreeing study processes. Solutions were achieved through careful planning and support from site based staff.

## Conclusion

Challenges in setting up and running a large diagnostic accuracy study can be overcome given adequate resource and planning. http://www.abdn.ac.uk/chart/gate.

